# Chronic Treatment of Ascorbic Acid Leads to Age-Dependent Neuroprotection against Oxidative Injury in Hippocampal Slice Cultures

**DOI:** 10.3390/ijms22041608

**Published:** 2021-02-05

**Authors:** Kyung Hee Lee, Un Jeng Kim, Myeounghoon Cha, Bae Hwan Lee

**Affiliations:** 1 Department of Dental Hygiene, Division of Health Science, Dongseo University, Busan 47011, Korea; kyhee@dongseo.ac.kr; 2 Department of Physiology, Yonsei University College of Medicine, Seoul 03722, Korea; mignon@yuhs.ac (U.J.K.); mhcha@yuhs.ac (M.C.); 3 Brain Korea 21 PLUS Project for Medical Science, Yonsei University College of Medicine, Seoul 03722, Korea

**Keywords:** ascorbic acid, antioxidant, aging, organotypic hippocampal slice culture, neuroprotection

## Abstract

Increased oxidative damage in the brain, which increases with age, is the cause of abnormal brain function and various diseases. Ascorbic acid (AA) is known as an endogenous antioxidant that provides neuronal protection against oxidative damage. However, with aging, its extracellular concentrations and uptake decrease in the brain. Few studies have dealt with age-related functional changes in the brain to sustained ascorbate supplementation. This study aimed to investigate the susceptibility of hippocampal neurons to oxidative injury following acute and chronic AA administration. Oxidative stress was induced by kainic acid (KA, 5 µM) for 18 h in hippocampal slice cultures. After KA exposure, less neuronal cell death was observed in the 3 w cultured slice compared to the 9 w cultured slice. In the chronic AA treatment (6 w), the 9 w-daily group showed reduced neuronal cell death and increased superoxide dismutase (SOD) and Nrf2 expressions compared to the 9 w. In addition, the 9 w group showed delayed latencies and reduced signal activity compared to the 3 w, while the 9 w-daily group showed shorter latencies and increased signal activity than the 9 w. These results suggest that the maintenance of the antioxidant system by chronic AA treatment during aging could preserve redox capacity to protect hippocampal neurons from age-related oxidative stress.

## 1. Introduction

Oxidative stress is caused by reactive oxygen species (ROS) and leads to structural and functional cellular changes in neurons, including apoptosis and necrosis [1]. Neurons are especially vulnerable to oxidative stress due to their high lipid content and high metabolic rates. Under physiological conditions, ROS are removed by a cellular antioxidant defense system that includes redox homeostasis and cellular signal transduction. Even though ROS are considered as damaging agents, ROS generation is initially necessary for cell function, as ROS act as intracellular messengers and serve as a platform for the transmission of physiological cellular redox signals in mitochondria and the cellular environment [2,3]. However, over-produced ROS lead to an accumulation of excess oxidant radicals that could damage the mitochondria and neuronal cells [4]. Increased ROS in the aging process progressively reduces the maintenance of tissue homeostasis and increases the likelihood of degenerative diseases. Studies on aging over the past several decades have shown that free radicals and oxidative stress increase with age and cause age-related increases in oxidative damage to many human cellular molecules [5]. These previous studies have implicated ROS as a source of oxidative damage to DNA, proteins, and lipids [6,7,8,9]. Mitochondria-generated ROS, as byproducts of mitochondrial respiration, are primary targets for oxidative damage and play an important role in aging [10,11]. Vitamins C and E are the main dietary antioxidants that protect erythrocytes from damage caused by ROS [12].

Ascorbic acid (AA), also known as vitamin C, is more highly concentrated in the brain than in other organs and plays an important role in neuronal differentiation and myelin formation [13]. For normal homeostasis of the nervous system, AA is considered to be the most important nutrient due to its crucial role in the brain’s antioxidant defense system responsible for scavenging reactive oxygen and nitrogen species produced during cellular metabolism [14,15]. High concentrations of AA have been observed predominantly in neuron-rich areas of the hippocampus and neocortex in the human brain [16], and the ascorbate content in neurons compared to glia appears to be significantly different [17]. Therefore, AA is very important for brain function and maintenance. Furthermore, many degenerative central nervous system diseases, including Alzheimer’s disease, multiple sclerosis, Parkinson’s disease, and Huntington’s disease [18], as well as psychiatric disorders, such as depression, anxiety disorders, and schizophrenia [13,14], are highly associated with AA deficiency. Brain levels of α-tocopherol, ascorbate, and glutathione have all been reported to decrease with aging, and the ascorbate level decrease was accompanied by decreased ascorbate synthesis and altered ascorbate transport characteristics [19]. These decreased ascorbate levels could either contribute to and/or result from the aging process. Interestingly, Michels et al. [20] demonstrated that declines in hepatic ascorbate in rats were associated with an age-related change in ascorbate uptake. Nevertheless, few studies have dealt with age-related functional changes in the brain to sustained ascorbate supplementation or the optical activity of neurons capable of restoring functional activity using optical imaging. 

To investigate the results according to age and AA treatment effects, the present study evaluated the neuroprotective properties of ascorbate treatment for various age-related oxidation states in hippocampal slices, as well as the difference between acute and chronic administration of ascorbate during aging. In addition, optical images were used to evaluate the functional role of ascorbate-rescued neurons after oxidative damage caused by kinetic acid (KA).

## 2. Results

### 2.1. Different Neuroprotection Effects of AA

**Figure 1:**
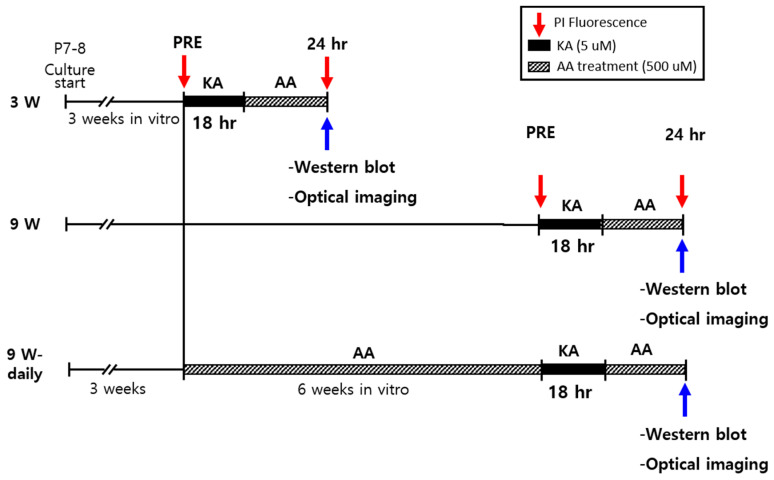
Experimental design and paradigm. Organotypic hippocampal slice cultures (OHSCs) were cultured for 3 w and 9 w. Slices were treated with propidium iodide (PI) 2 h before 5 µM kinetic acid (KA) treatment for all cultures (PRE). All received KA treatment except for the normal controls in each experimental group. After 18 h exposure to KA, the medium containing KA was changed to a fresh culture medium containing 500 µM ascorbic acid (AA). PI uptake, Western blot, and electrophysiological studies were conducted 24 h after AA treatment. Experimental groups were divided by culture time and treatment type, such as 3 w, 9 w, and 9 w-daily. In the 9 w-daily group, 500 µM AA was added to the culture medium for 6 w before studies began.

At 3 w or 9 w after hippocampal slice culture, 5 µM KA was applied for 18 h to induce oxidative injury and was included in fresh medium (vehicle group). AA with medium was replaced for 24 h after KA treatment (AA treatment). For the 9 w experiment groups, one was treated daily with AA from week 3 to week 9 (9 w-daily), and the other was treated only with medium from week 3 to week 9 (9 w). The experimental groups and the overall design are presented in Figure 1. 

**Figure 2:**
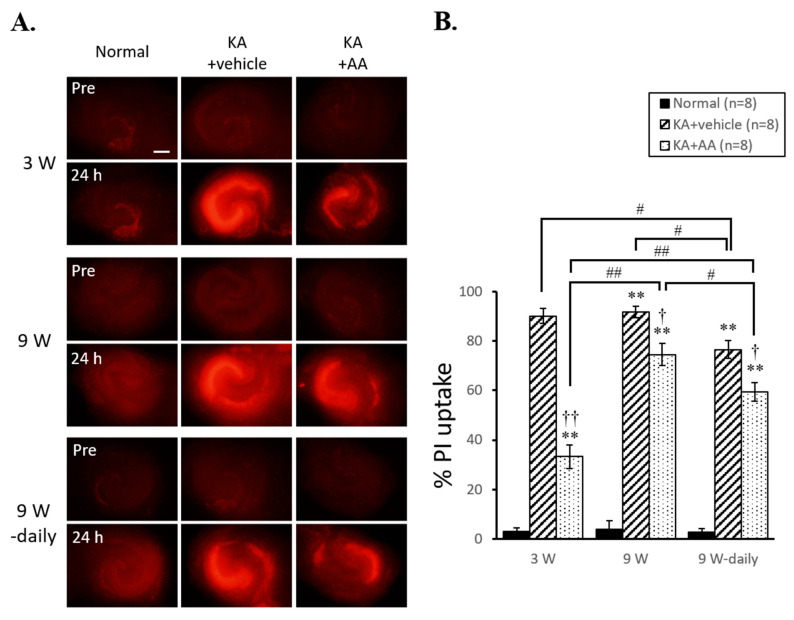
Neuroprotective effects of AA against KA-induced oxidative injury in hippocampal neurons. (**A**) Representative PI fluorescence images in OHSCs before (Pre) and 24 h after AA-treatment following oxidative injury. Representative images of PI uptake, as a marker of cell death, showed the effects of AA treatment at different time points. AA-only treatment (500 µM) showed no toxic effect in KA-untreated normal OHSCs (9 w-daily Normal). (**B**) KA treatment induced progressive cell death in the hippocampus compared to the normal group at each time point. After 24 h of AA treatment, PI signals in the CA3 area of the hippocampus were significantly reduced in the treated groups compared to no-treatment (vehicle) groups at each time point. After chronic AA treatment (9 w-daily), there was less cell death following the KA insult compared to the 9 w vehicle group, and the 9 w-daily AA treatment group also showed a significant decrease in the level of PI uptake compared to the 9 w AA treatment group. ** *p* < 0.01 compared to normal; † *p* < 0.05, †† *p* < 0.01 compared to vehicle; # *p* < 0.05, ## *p* < 0.01 comparing different time points: two-way ANOVA followed by Tukey’s *post hoc* comparison. Scale bar: 500 µm.

Figure 2 shows the different neuroprotective effects of AA in 3 w, 9 w, and 9 w-daily organotypic hippocampal slice cultures (OHSCs) before (Pre, 2A upper line) and 24 h after oxidative injury. KA treatment (5 µM) led to progressive cell death in the CA3 area of the hippocampus after 24 h (24 h, 2A bottom line). Hippocampal neurons in the 3 w KA + AA group showed reduced propidium iodide (PI) uptake compared to the 3 w KA + vehicle group. Similar to the results of 3 w group, the 9 w KA + AA and 9 w-daily KA + AA groups also showed decreased PI uptake compared to the 9 w KA + vehicle and 9 w-daily KA + vehicle groups. These results indicate the neuroprotective effects of AA after KA exposure. Statistical analysis of neuroprotective effects of AA is shown in Figure 2B. The 3 w KA + vehicle and KA + AA PI uptake values were significantly increased compared to the 3 w normal PI uptake (normal: 3.01 ± 1.58, KA + vehicle: 90.03 ± 3.02, KA + AA: 33.33 ± 4.75). In addition, 9 w of KA + vehicle and KA + AA and 9 w-daily KA + vehicle and KA + AA value were significantly increased (9 w normal: 3.80 ± 2.01, KA + vehicle: 91.83 ± 2.33, KA + AA: 74.46 ± 4.50; 9 w-daily normal: 2.73 ± 1.38, KA + vehicle: 76.54 ± 3.68, KA + AA: 59.37 ± 3.78). Moreover, 9 w KA + AA group showed significantly increased cell death compared to 3 w KA + AA group. These results indicate that the aging group (9 w) was more vulnerable compared to the young group (3 w) to cell death and less sensitive to the protective effect of AA treatment following an oxidative injury. In addition, 9 w-daily KA + vehicle and 9 w-daily KA + AA groups showed significantly reduced PI uptake compared to 9 w KA + vehicle and 9 w KA + AA groups. These results indicate that chronic AA treatment protects hippocampal neurons from KA-induced oxidative injury and that prolonged antioxidant treatment during the aging process has a neuroprotective effect. 

### 2.2. Activation of Antioxidant Signals by AA Treatment in Aging

**Figure 3:**
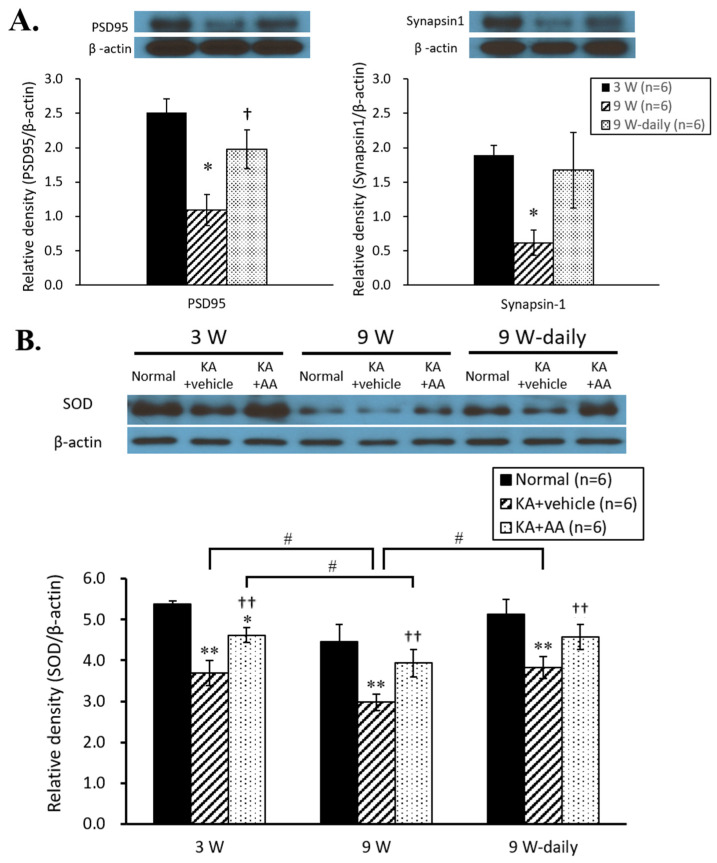

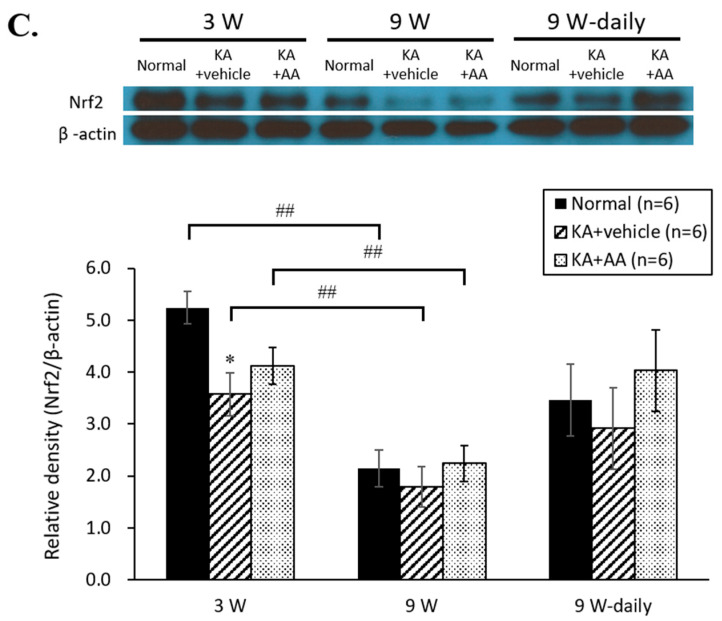
(**A**) Changes in synaptic protein expression by culture time of OHSCs. PSD 95 and synapsin-1 expression levels significantly reduced at 9 w compared to 3 w. In the chronic AA treatment group, the expression levels of PSD 95 and synapsin-1 proteins showed a significant difference between 9 w and 9 w-daily groups. (**B**) Western blot analysis of SOD and Nrf2 in OHSCs following oxidative injury. The expression of SOD in the vehicle group was significantly reduced compared to the normal group. The expression of SOD significantly increased 24 h after 500 µM AA treatment in the 3 w and 9 w groups, compared to the vehicle group. The chronic AA treatment (9 w-daily) group showed higher SOD expression than the 9 w group. (**C**) The expression level of Nrf2 increased in the AA treatment group compared to the vehicle group at 3 w. The 9 w group showed no difference in Nrf2 expression between the AA treatment and vehicle groups. However, Nrf2 expression was significantly decreased in both AA treatment and vehicle groups at 9 w compared to 3 w. In 9 w-daily groups, Nrf2 expression tended to increase compared to 9 w. The horizontal axis indicates each experimental group (3 w, 9 w, and 9 w-daily AA treatment), and the vertical axis represents the normalized level of each protein expression (ratio of each antibody expression/β-actin expression). * *p* < 0.05, ** *p* < 0.01 compared to normal, † *p* < 0.05, †† *p* < 0.01 compared to vehicle, # *p* < 0.05, ## *p* < 0.01 comparing different time points: two-way ANOVA followed by Tukey’s post hoc comparison.

In our aging model of OHSCs with time in culture, we observed age-related changes in the hippocampus. Synapsin-1 and PSD 95, pre- and post-synaptic components implicated in dendritic spine formation and neurotransmission, were observed to confirm the aging statuses of each culture condition. PSD 95 expression was significantly reduced in the 9 w group compared to 3 w group, while the 9 w-daily group showed significantly higher PSD 95 expression than the 9 w group. For synapsin-1, the 9 w group showed markedly lower expression levels than the 3 w and 9 w-daily groups. The expression levels of synapsin-1 and PSD 95 in the 9 w group was markedly reduced compared to the 3 w group, which indicated the age-related phenomena within the hippocampus in our aging model of OHSCs. As for the chronic AA treatment group, the expression levels of both synapsin-1 and PSD 95 proteins showed significant differences between the 9 w and 9 w-daily groups (Figure 3A). These results indicated that chronic AA treatment conserved the decreases in synaptic ability associated with neurodegeneration in aging.

To observe the antioxidant role of prolonged AA treatment on cell survival, we examined the expression of superoxide dismutase (SOD) and Nrf2, which are known to be factors in ROS-related cell survival signaling. At 3 w, the expression levels of SOD protein significantly increased in the KA + AA group compared to KA + vehicle group. In the aging model, the 9 w KA + AA group showed significantly higher SOD levels than the KA + vehicle group. Compared to the 3 w KA + AA group, the levels of SOD expression in the 9 w KA + AA group were significantly reduced. Moreover, SOD protein significantly increased in the 9 w-daily KA + AA group compared to the KA + vehicle group. The 9 w-daily KA + vehicle group also showed significantly increased SOD levels compared to the 9 w KA + vehicle group (Figure 3B). Nrf2 expression reflects the susceptibility of the brain to the damaging effects of ROS: the level of Nrf2 expression diminished with age, and consequently, its neuroprotective effect decreased. The expression levels of Nrf2 protein decreased in the vehicle group compared to the normal group at 3 w. With aging, the 9 w group showed no difference in Nrf2 expression between KA + AA and KA + vehicle groups. Compared to 3 w, Nrf2 expression was significantly decreased at 9 w for both KA + AA and KA + vehicle groups. In the 9 w-daily group, Nrf2 expression tended to be higher compared to the 9 w treatment groups (Figure 3C).

### 2.3. Effect of AA on Neuronal Activity

**Figure 4:**
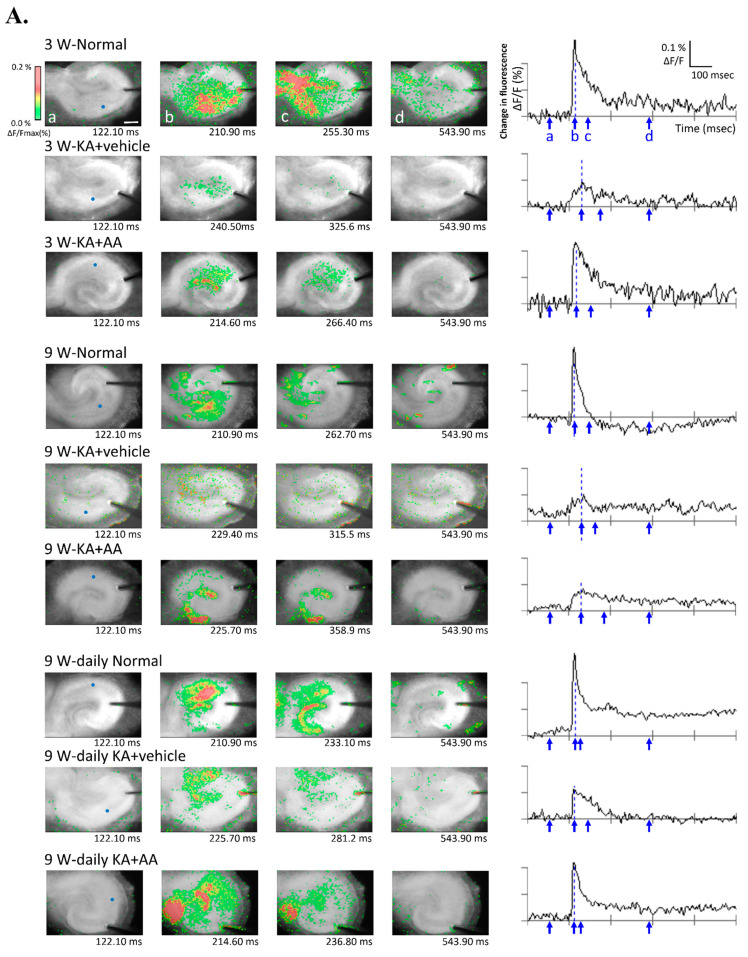

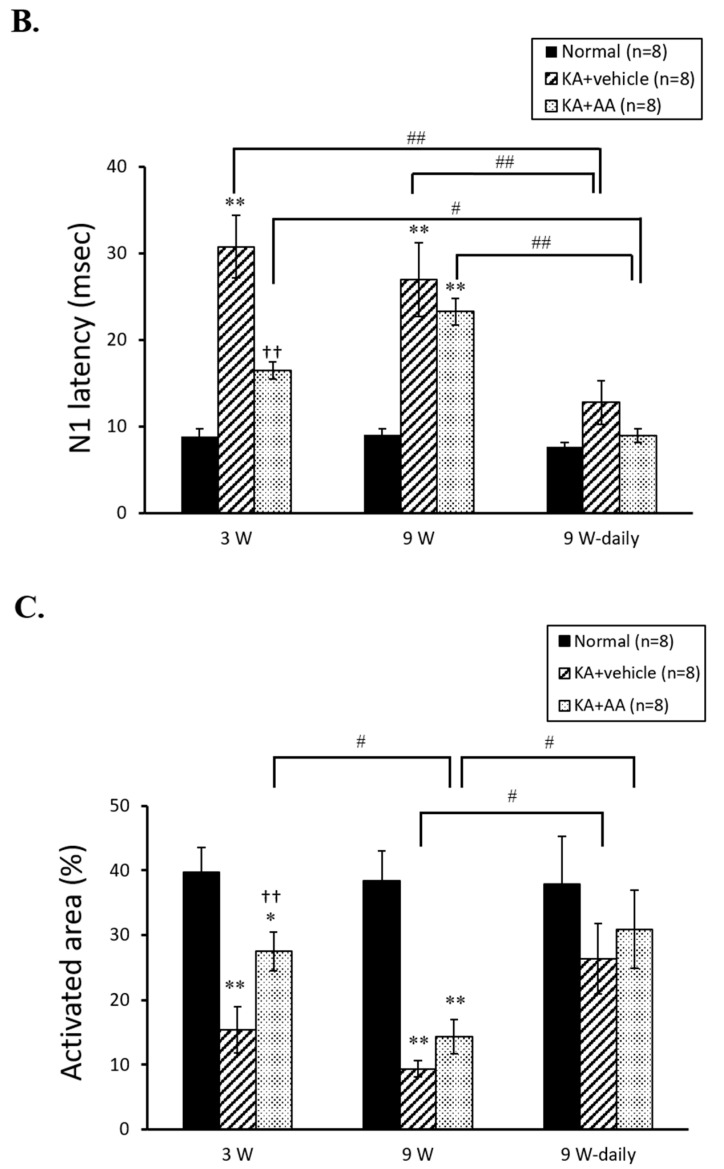
Optical recordings were conducted with a voltage-sensitive dye (VSD) in hippocampal slices to assess AA treatment effects. (**A**) Representative pseudo-color optical images show the evoked excitatory neuronal signals and typical spatiotemporal changes that were observed in OHSCs after electrical stimulation. Each frame of the pseudo-color image (left side) is presented by an arrow below the wave form (right side) at each time point (a, before stimulation; b, peak of N1; c, half of the N1; d, post stimulation). The X-axis shows the time (msec), and the y-axis shows the percent change in fluorescent intensity (% ΔF/F). The blue grid line shows the peak of the negative (N1) point, which was used to quantify the N1 latency. A colored scale (color calibration) shows the changes in optical signals (upper-left side). Signal transmission indicating spatiotemporal changes showed an increase in neuronal activity through long-lasting depolarization. The KA + vehicle groups at each time point showed a few signs of activation, only in the focal stimulation area. Spatiotemporal changes, reflecting the distribution of neuronal activity, increased after AA treatment. (**B**) Comparisons of optical signals in the different experimental groups by latency, an indication of basal synaptic transmission. Latencies were significantly delayed in the vehicle groups compared to normal groups at each time point. The latency of the optical signals was decreased in AA-treated hippocampal slices compared to the vehicle group at 3 w. For chronic AA treatment, there was a significant difference between the 9 w and 9 w-daily groups. (**C**) Quantification of pseudo-color activated areas that indicate neuronal activities through neural propagation. The activation area was decreased in the vehicle groups compared to the normal groups at each time point. In particular, the chronic AA-treated 9 w-daily group showed a significantly greater increase in activation area compared to the acute 9 w group. * *p* < 0.05, ** *p* < 0.01 compared to normal, †† *p* < 0.01 compared to vehicle, # *p* < 0.05, ## *p* < 0.01 comparing different time points: two-way ANOVA followed by Tukey’s post hoc comparison. Scale bar: 500 µm.

We performed optical imaging to observe cellular activities to visualize the active area based on the overall data and provide information on neuronal activation using a voltage-sensitive dye (VSD). Typical signal transmission and the spatial distribution of the cellular response after electrical stimulation in different age-related groups are shown in Figure 4A. As shown in Figure 4A, KA-untreated hippocampal neurons (normal groups at 3 w, 9 w, and 9 w-daily) demonstrated typical spatiotemporal changes using Schaffer collateral/commissural stimulation. However, limited activation accompanied by synaptic propagation was observed in the KA + vehicle group of OHSCs. In contrast, more activation (indicating more propagation) was observed in the KA + AA group compared to the KA + vehicle group in each age-related group.

N1 latency, a sign of synaptic activation, increased in both KA + vehicle and KA + AA groups compared to both 3 w and 9 w normal groups by optical signals. The statistical analysis of the neuronal activity of AA is shown in Figure 4B. At 3 w, the AA treatment group showed a significantly reduced latency compared to the KA + vehicle group (normal: 8.84 ± 0.91, KA + vehicle: 30.75 ± 3.66, KA + AA: 16.45 ± 0.97). In 9 w, there was no difference between KA + vehicle and KA + AA groups in N1 latency (9 w normal: 9.05 ± 0.64, KA + vehicle: 26.93 ± 4.27, KA + AA: 23.23 ± 1.56). Meanwhile, N1 latencies in the 9 w-daily groups were significantly shorter than those in the 9 w KA + vehicle and KA + AA groups (9 w-daily normal: 7.66 ± 0.46, KA + vehicle: 12.75 ± 2.52, KA + AA: 8.94 ± 0.81). These results showed that the chronic treatment AA (9 w-daily) in the aged group affected the neuronal activity as an indication of survival neuronal function (Figure 4B). In the activation area in Figure 4C, the KA + AA group showed significantly activated areas compared to the KA + vehicle group at 3 w (normal: 39.70 ± 3.81, KA + vehicle: 15.40 ± 3.58, KA + AA: 27.52 ± 3.00). In 9 w OHSCs, few activated areas were observed around the stimulating electrode in both the KA + vehicle and KA + AA groups (9 w normal: 38.41 ± 4.64, KA + vehicle: 9.39 ± 1.31, KA + AA: 14.38 ± 2.61). There was no difference between the 9 w-daily groups (9 w-daily normal: 37.81 ± 7.44, KA + vehicle: 26.37 ± 5.44, KA + AA: 30.90 ± 6.02). However, chronic AA treatment in the 9 w-daily KA + AA group elicited significantly more activated areas than the 9 w KA + AA group. In addition, the 9 w-daily KA + vehicle group showed significantly larger activated areas compared to the 9 w KA + vehicle group (Figure 4C). Therefore, synaptic propagation was better preserved and maintained after chronic AA treatment in the 9 w-daily group, and this group showed less vulnerability to oxidative injury compared to other 9 w groups.

## 3. Discussion

Providing cells with AA as an exogenous antioxidant retards their uptake of endogenous antioxidants. In agreement with previous research [21], AA treatment elicited significant reductions in neuronal cell death, compared to vehicle, through a reduction in ROS and oxidative stress by the activation of antioxidant pathways. However, the negative effect of oxidative decay caused by ROS is considered to be an irreversible progression in the biology of aging [22]. Research has shown that the number of neurons and astrocytes remain constant over 21 days in vitro (DIV, 3 w) among OHSCs by cell density analysis and that the density of synaptic contacts within 21 DIV OHSCs is two times higher than those in 7 DIV OHSCs [23]. Moreover, DIV 21 cultures have been found to show similarities to features of the acute slice at P21 [24]. Synapses formed in OHSCs arise from neurons that survive explanation, and synaptic differences in OHSCs according to culture time have been observed, with significant reductions in PSD 95 and evoked synaptic activity at 67-76 DIV [25]. In line with the study by Mielke et al. [25], we also observed significant decreases in PSD 95 and synapsin-1 expressions, which were assessed to reflect age-related phenomena within the hippocampus in our aging model of OHSCs. 

Age-dependent changes in brain homeostasis and function occur gradually, particularly by the combination of ROS and the aging brain’s impaired ability to repair increased oxidative stress damage [26]. Although we observed decreased neuronal cell death with acute AA treatment after 3 w and 9 w, compared to vehicle, PI uptake quantitation, as an indicator of cell death, was significantly different (based on age) between the 3 w and 9 w AA treatment groups. The 9 w group showed more vulnerability to oxidative injury and cell death compared to the 3 w group. Aging of the brain was also associated with an increase in seizure susceptibility, as well as with seizure-induced neuronal damage, after KA-induced oxidative injury [27,28]. While Siqueira et al. [19] reported that 2 weeks of chronic supplementation with ascorbate was unable to protect the hippocampus from age-related oxidative damage (H_2_O_2_), we observed that the 9 w-daily vehicle and 9 w-daily AA treatment groups showed significantly greater reductions in cell death than did the 9 w AA treatment group. As a consequence of aging, decreased ascorbate synthesis or altered ascorbate transport characteristics [19] may explain this reduced neuroprotective effect of acute AA treatment in the 9 w group compared to chronic AA treatment in the 9 w-daily group. We hypothesized that chronic AA treatment may lead to persistent regulation of reactions against increased reactive oxygen and nitrogen species with various target molecules during aging, a period during which the antioxidant system appears to be less functional in the brain. Mild prooxidant activity can enhance antioxidant defense systems through Nrf2 signaling [29,30,31]. In this regard, chronic treatment of AA, which is not only able to scavenge free radicals but also maintain an optimal ROS flow, may upregulate cytoprotective enzymatic antioxidants, thus improving neuronal survival. Chronic treatment of AA might act as a parahormetic phytochemical, which can be attributed to both antioxidant and mild prooxidant activities that affect the intracellular antioxidant defense system. As part of the antioxidant defense system, any decrease in SOD and Nrf2 levels would result in increased ROS [32]. In our Western blot analysis, the expressions of SOD and Nrf2 significantly decreased in the 9 w groups compared to the 3 w groups. Imbalances in ROS production due to impaired expression of SOD and decreased levels of antioxidant molecules occurred with aging, as well as in neurodegenerative diseases [33]. Similar to the PI results, chronic AA treatment (9 w-daily AA treatment group) significantly increased SOD levels. The neurons in the 9 w group suffered more severe attacks by free radicals compared to the neurons with chronic AA treatment, and AA supplementation might have scavenged free radicals from plasma directly. The present study indicates that the aging brain is highly susceptible to hippocampal cell loss by KA-induced oxidative stress, and that chronic AA treatment can aid the cellular antioxidant system against oxidative injury.

To assess neuronal survival and functional property, optical imaging of VSD was used to examine synaptic changes and strength in OHSCs. Optical imaging data represent the visualization of electrical signals from the population activity of postsynaptic neurons in the activated area as a distribution map of stimulus-induced activities [34]. Here, the differences in activities of surviving neurons were examined between 3 w and 9 w slice cultures that underwent AA treatment after oxidative injury. The 9 w group showed increased latencies of optical signals and less activated areas compared to the 3 w group, indicating reduced functionality of surviving neurons. This result may be related to decreased uptake of radiolabeled ascorbate in aged rats [19], as well as to a decline in sodium-dependent ascorbate transport during the aging process [20]. Siqueria et al. [19] reported a 40% decrease in radiolabeled ^14^C-ascorbate uptake in 11-month-old rats compared to 4-month-old rats, and Michels et al. [20] described an age-related reduction in the plasma transport of dietary ascorbate. Neurons have a 10-fold-higher level of ascorbate than the glia, making them more sensitive to ascorbate deficiency and any reduction in total antioxidant capacity in an older hippocampus [19]. The VSD activation area is dependent on the spatial distribution of cellular responses to electrical stimulation. In this study, we demonstrated enhanced synaptic transmission resulting from chronic AA treatment in older 9 w OHSCs after oxidative injury. To our knowledge, there are no reports that chronic AA supplementation protects hippocampal cells in the brain from the age-related increases in susceptibility to oxidative damage. Therefore, with the optical recording results demonstrating better survival of hippocampal neurons and their connections, chronic AA supplementation may not only be neuroprotective, but also help restore the endogenous antioxidant system with advancing age.

## 4. Materials and Methods

### 4.1. Organotypic Hippocampal Slices Cultures (OHSCs) 

Animal experiments were approved by the Institutional Animal Care and Use Committee of Yonsei University Health System (permit no.: 2018-0095, approval date: 8 May 2018). Postnatal Sprague-Dawley rats (6–7 d) were used according to the method of Stoppini et al. [35]. We used 45 rat pups, and 40 hippocampal slices were used in each group (one batch contained five hippocampal slices, *n* = 8 batches). Hippocampi were dissected and placed in Gey’s salt solution (Sigma, Saint Louis, MO, USA) with glucose (6.5 mg/mL). Slices (350 µm thick) were cut parallel to the transverse axis of the hippocampus using a chopper (McIlwain tissue chopper; Mickle Laboratory Engineering Ltd., Surrey, UK). Millicell culture inserts (Millipore, Billerica, MA, USA) containing five slices were cultured in 6-well plates with medium (50% opti-MEM, 25% HBSS, 25% horse serum, 6.5 mg/mL glucose, pH adjusted to 7.2) for 3 w or 9 w. 

### 4.2. Propidium Iodide (PI) Staining 

To induce oxidative injury, 5 µM KA (Sigma) was applied for 18 h and was included with fresh medium in 3 w or 9 w after hippocampal slice culture (KA + vehicle). AA with medium was replaced for 24 h with 5 ug/mL of propidium iodide (PI) (5 µg/mL, Sigma), which was added to the culture medium (KA + AA). In chronic AA treatment, OHSCs were maintained in culture medium with 500 µM AA for 6 w before KA exposure (9 w-daily). As previously described [36], neuronal death was assessed by quantifying the fluorescence intensity of PI. This was based on the principle that PI is impermeable to normal plasma membranes and cells, and when cells are damaged, they migrate to the nucleus, where they form a complex with DNA, making the nucleus fluoresce. PI uptake images were captured with a fluorescence microscope digital camera (BX-51, Olympus, Tokyo, Japan) and quantified with the MetaMorph Imaging System (Universal Image Co, Downingtown, PA, USA). PI uptake was measured before (Pre) and 24 h after the application of drugs, and the value of the measured fluorescence area was expressed [(24 h − Pre/full kill − Pre) × 100] as the % PI uptake. N-methyl-D-aspartate (NMDA) (100 µM, Sigma) was applied to induce fulminant death of pyramidal neurons (full kill) at the termination of each experiment [37,38]. AA treatment alone (500 µM) showed no toxic effect in KA-untreated normal OHSCs (Normal group) at 3 w or 9 w.

### 4.3. Western Blot Analysis

Homogenized slice lysates were prepared with lysis buffer (PRO-PREP^TM^, Intron Biotechnology, Burlington, MA, USA). Proteins were resolved by SDS-PAGE (100 µg/lane) using 10% (*w*/*v*) polyacrylamide gels and transferred to PVDF membranes (Millipore). Membranes were incubated with antibodies against anti-PSD 95 (1:5000, Abcam, Cambridge, MA, USA); anti-synapsin I (1:3000, Abcam); superoxide dismutase (SOD) (1:7500, Abcam); and Nrf2 (1:5000, Abcam) for 2 h at room temperature. As a loading control, membranes were also probed with anti-β-actin antibody (1:10,000, Abcam). The reaction was developed with an enhanced chemiluminescence Western blot analysis system (ECL, GE Healthcare, Marlborough, MA, USA). Signal intensities were analyzed using a gel-scanning integrated optical density software program (Multi-gauge 3.0, Fuji film, Tokyo, Japan).

### 4.4. Optical Recording

Hippocampal slices (*n* = 8) were stained with a voltage-sensitive dye (VSD) solution (Di-2-ANEPEQ, JPW114; Invitrogen, Carlsbad, CA, USA) [39]. Briefly, 0.2 mM Di-2-ANEPEQ staining solution was applied to the OHSCs in the plexiglass mesh-attached ring. The OHSCs were maintained in a humidified chamber with a mixture of O_2_ and CO_2_ gases for 25 min. The OHSCs were rinsed with artificial cerebrospinal fluid (ACSF: composition in mM: NaCl, 124; NaHCO_3_, 26; glucose, 10; KCl, 3; CaCl_2_, 2; MgCl_2_, 1; HEPES, 10; pH 7.4) by dipping them through the plexiglass mesh-attached ring and kept for at least 1 h to be recovered before being used for the experiment. A stained hippocampal slice was attached to the chamber glass, which was pre-coated with 0.01% polyethylenimine for approximately 1 h and rinsed with distilled water. Stained OHSCs were stabilized in the recording chamber, which was continuously perfused with ACSF at 31 °C bubbled with mixed 95% O_2_ and 5% CO_2_ gases. The Schaffer collateral (SC)/commissural pathway was stimulated with a bipolar electrode (CBBPE75, FHC Inc, Bowdoin, ME, USA). Synaptic activity was observed using an optical imaging system (MiCAM02, Brain Vision Inc., Tsukuba, Japan) with a high-speed CCD camera. Optical images were recorded with 512 frames (1890 ms total) for the test stimulation and acquired at a sampling rate of 3.7 ms per frame. Trials were conducted every 20 s (0.05 Hz). Fluorescence intensity [ΔF/F: the change in the intensity of fluorescence (ΔF) in each pixel relative to the initial intensity of fluorescence (F)] was used to assess fractional changes in the amount of VSD fluorescence. Activated areas were determined by averaging images (spatial filter: 3 × 3 pixels, cubic filter: 3 × 3 pixels). Acquisition and analysis software (BV-Analyzer, Brain Vision Inc.) was used to display and analyze the optical images.

### 4.5. Statistical Analysis

Statistical analyses were performed using GraphPad Prism (GraphPad Software, San Diego, CA, USA). Data are expressed as means ± standard errors of the mean (SEM). The data were evaluated by two-way analysis of variance (ANOVA) followed by Tukey’s multiple-range post hoc comparisons. Differences were considered significant when *p*-values were less than 0.05.

## 5. Conclusions

In this study, we demonstrated that chronic AA treatment has a neuroprotective influence on KA-induced oxidative stress in an aging hippocampus model. Based on PI staining, chronic AA treatment also reduced age-related neurotoxicity (9 w-daily AA treatment), and this was similar to its influence on oxidative injury in younger hippocampi (3 w AA treatment). Western blot analysis demonstrated that chronic AA treatment activated SOD, a well-known detoxifying enzyme, compared to acute AA treatment in the 9 w group. Therefore, we have shown that chronic AA treatment increases antioxidant activity and decreases neuronal cell death in this model. Using optical signals to observe the functional recovery of surviving neurons, AA treatment decreased latencies of the optical signal, indicating an increase in effective neuronal transmission. These results suggest that surviving neurons, protected by chronic AA treatment, show enhanced functional recovery in this aging model.

## Data Availability

Not Applicable.
